# FLT3-ITD induces expression of Pim kinases through STAT5 to confer resistance to the PI3K/Akt pathway inhibitors on leukemic cells by enhancing the mTORC1/Mcl-1 pathway

**DOI:** 10.18632/oncotarget.22926

**Published:** 2017-12-04

**Authors:** Keigo Okada, Ayako Nogami, Shinya Ishida, Hiroki Akiyama, Cheng Chen, Yoshihiro Umezawa, Osamu Miura

**Affiliations:** ^1^ Department of Hematology, Graduate School of Medical and Dental Sciences, Tokyo Medical and Dental University, Tokyo, Japan

**Keywords:** AML, FLT3, PI3K, STAT5, Pim kinases

## Abstract

FLT3-ITD is the most frequent tyrosine kinase mutation in acute myeloid leukemia (AML) associated with poor prognosis. We previously reported that activation of STAT5 confers resistance to PI3K/Akt inhibitors on the FLT3-ITD-positive AML cell line MV4-11 and 32D cells driven by FLT3-ITD (32D/ITD) but not by FLT3 mutated in the tyrosine kinase domain (32D/TKD). Here, we report the involvement of Pim kinases expressed through STAT5 activation in acquisition of this resistance. The specific pan-Pim kinase inhibitor AZD1208 as well as PIM447 in combination with the PI3K inhibitor GDC-0941 or the Akt inhibitor MK-2206 cooperatively downregulated the mTORC1/4EBP1 pathway, formation of the eIF4E/eIF4G complex, and Mcl-1 expression leading to activation of Bak and Bax to induce caspase-dependent apoptosis synergistically in these cells. These cooperative effects were enhanced or inhibited by knock down of mTOR or expression of its activated mutant, respectively. Overexpression of Mcl-1 conferred the resistance on 32D/ITD cells to combined inhibition of the PI3K/Akt pathway and Pim kinases, while the Mcl-1-specific BH3 mimetic A-1210477 conquered the resistance of MV4-11 cells to GDC-0941. Furthermore, overexpression of Pim-1 in 32D/TKD enhanced the mTORC1/Mcl-1 pathway and partially protected it from the PI3K/Akt inhibitors or the FLT3 inhibitor gilteritinib to confer the resistance to PI3K/Akt inhibitors. Finally, AZD1208 and GDC-0941 cooperatively inhibited the mTORC1/Mcl-1 pathway and reduced viable cell numbers of primary AML cells from some FLT3-ITD positive cases. Thus, Pim kinases may protect the mTORC1/4EBP1/Mcl-1 pathway to confer the resistance to the PI3K/Akt inhibitors on FLT3-ITD cells and represent promising therapeutic targets.

## INTRODUCTION

FMS-like tyrosine kinase 3 (FLT3) is a receptor-tyrosine kinase expressed on hematopoietic progenitors and regulates early steps of hematopoietic progenitor cell proliferation, survival, and differentiation [1–3]. Internal tandem duplication (ITD) mutations in the juxtamembrane domain of FLT3 (FLT3-ITD) are the most frequent kinase mutations in acute myeloid leukemia (AML), occurring in 25–30% of cases. Point mutations within the tyrosine kinase domain (FLT3-TKDs), such as the most frequent D835Y mutation, are also found in 5–10 % of patients with AML. It has been well established that FLT3-ITD confers a poor prognosis due to intrinsic therapy resistance with lower complete response rates and higher relapse rates leading to inferior disease-free and overall survivals, which is particularly significant in AML with higher mutant to wild-type allelic ratio and the insertion site of ITD in the tyrosine kinase domain [4, 5]. Although a multi-targeted kinase inhibitor, midostaurin, has shown a significant effect in combination with the standard chemotherapy against FLT3-mutated AML and was recently approved by the US Food and Drug Administration (FDA) for its treatment [6, 7], clinical trials with specific FLT3 tyrosine kinase inhibitors alone have so far given only limited successes [3], at least partly because of emergences of resistance mutations after sustained FLT3-ITD inhibition in the case of FLT3-specific inhibitor AC-220 (quizartinib) [8].

FLT3-ITD as well as FLT3-TKD stimulates the various downstream signaling pathways that are normally activated by ligand-stimulated FLT3, such as the PI3K/Akt/mTOR and MEK/ERK pathways, thus leading to survival and proliferation of model hematopoietic cell lines or causing myeloproliferative disorders in various murine models [1–3]. In addition, FLT3-ITD but not FLT3-TKD strongly activates STAT5, which contributes to enhanced transforming potentials of FLT3-ITD as compared with FLT3-TKD in cellular and animal models. [9–11]. The serine/threonine kinase mTOR is mainly activated downstream of the PI3K/Akt pathway and forms the two multi-protein complexes, mTORC1 and mTORC2, to regulate various cellular events, such as proliferation, apoptosis, and autophagy [12, 13]. Importantly, mTORC1 plays a critical role in regulation of cap-dependent translation by phosphorylating 4EBP1 to release it from the mRNA m^7^-GTP cap-binding protein eIF4E, thus allowing its interaction with the scaffolding protein eIF4G to initiate the formation of the translation-initiating complex eIF4F. This mechanism is important for the translation of mRNAs containing long 5’-UTRs with a high G+C content, such as those for Mcl-1, c-Myc, and cyclin D1 [14–16]. Mcl-1 is a highly-unstable anti-apoptotic Bcl-2 family member playing a crucial role in survival of hematopoietic progenitor cells and various malignant hematopoietic cells including AML cells [17]. The PI3K/Akt signaling pathway plays an important role in both normal and malignant hematopoiesis and represents a promising target for leukemia therapy [13, 18]. Thus, a large number of pharmacological inhibitors against PI3K and Akt have been developed and under preclinical or clinical studies as well as in clinical usage, such as GDC-0941 (pictilisib) and MK-2206, potent and selective inhibitors for the class I PI3Ks and all Akt isoforms, respectively, showing promising effects in preclinical studies [19, 20]. However, only modest effects on AML cells have been obtained at least partly due to the resistance of the downstream mTOR pathway to these inhibitors [18]. We have previously found that FLT3-ITD confers resistance to GDC-0941 and MK-2206 by protecting the mTOR pathway through the robust STAT5 activation to maintain the expression level of Mcl-1 [21].

The Pim family of proteins, composed of Pim-1, Pim-2, and Pim-3, are highly conserved and homologous serine/threonine kinases involved in cell survival, proliferation, and apoptosis [16, 22–24]. Although these kinases are ubiquitously expressed, Pim-1 and Pim-2 are predominantly expressed in hematopoietic cells, while Pim-3 is highly expressed in kidney, breast and brain cells. Two isoforms of Pim-1 with sizes of 34 and 44 kDa with comparable kinase activities are generated by translation of its mRNA from alternative initiation sites, while three isoforms of Pim-2 with 34, 37 and 40 kDa are similarly generated. Pim kinases are highly redundant and regulate the activity of their substrates involved in regulation of cap-dependent protein translation, cell survival, cell cycle, and Myc-dependent transcription [16]. Recent studies have implicated Pim kinases in enhancement of the mTORC1 pathway to upregulate cap-dependent translation in parallel with the PI3K/Akt pathway [24]. Because these kinases are constitutively active and short lived, their regulation mainly occurs primarily via transcription, translation, and protein stabilization regulating their expression levels. In hematopoietic cells, their expression is mainly regulated transcriptionally through the JAK/STAT pathway downstream of the hematopoietic cytokine receptor [22, 23, 25]. In particular, STAT5 has been demonstrated to transcriptionally upregulate expression of both Pim-1 and Pim-2 [26, 27]. Pim-1 and Pim-2 are frequently overexpressed in hematological malignancies, such as AML, chronic myeloid leukemia, and multiple myeloma, and implicated in their pathogenesis through various mechanisms, including upregulation of the mTORC1 pathway [16, 24]. In AML, it has been reported that Pim kinases are expressed at high levels in FLT3-ITD positive cases most likely through activation of STAT5 [3, 28, 29]. Pim knock out studies have shown that mice deficient in all three Pim kinases are viable and fertile, supporting the tolerability of pan-Pim kinase inhibition. Thus, various pan-Pim kinase inhibitors, such as AZD1208 [30] and PIM447 [31], as well as Pim-1-specific inhibitors have been developed and under evaluation in clinical trials for various hematological and other malignancies [22–24].

Because Pim kinases have been reported to be expressed downstream of STAT5 activation in FLT3-ITD-positive AML cells and implicated in upregulation of the mTORC1 pathway in various cell types, we have addressed in the present study the possible involvement of Pim kinases in acquisition of the resistance to PI3K/Akt pathway inhibitors by FLT3-ITD-expressing cells we have previously reported [21]. Using the model hematopoietic 32Dcl3 cell line expressing FLT3-ITD (32D/ITD) or FLT3-TKD (32D/TKD) and the FLT3-ITD-positive AML cell line MV4-11, we demonstrate that the pan-Pim kinase inhibitor AZD1208 or PIM447 downregulated the mTORC1/Mcl-1 pathway cooperatively with the PI3K/Akt pathway inhibitors in FLT3-ITD-expressing cells and abrogated the resistance of these cells to the PI3K/Akt inhibitors to induce apoptosis, while the resistance was conferred on FLT3-TKD-expressing cells by overexpression of Pim-1. Further studies have revealed that the Pim kinases partially protect the mTORC1 pathway when the PI3K/Akt pathway is inhibited to mitigate downregulation of Mcl-1 expression to maintain survival of FLT3-ITD-expressing cells, including primary AML cells.

## RESULTS

### Inhibition of Pim kinases abrogates the resistance to PI3K/Akt pathway inhibitors conferred through robust STAT5 activation by FLT3-ITD

**Figure 1:**
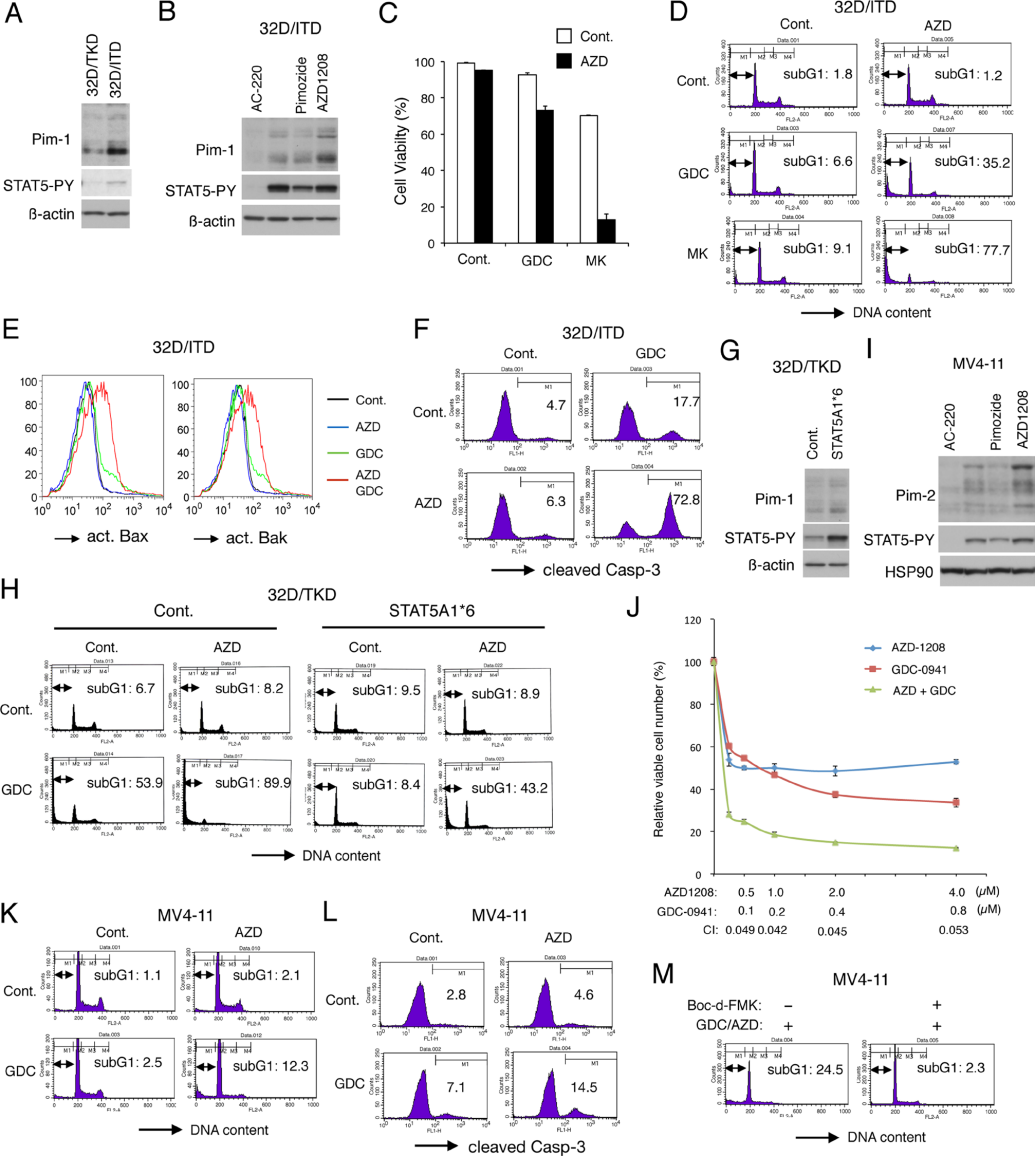
Inhibition of Pim kinases abrogates the resistance to PI3K/Akt pathway inhibitors conferred by robust STAT5 activation by FLT3-ITD (**A**) 32D/TKD or 32D/ITD cells were lysed and subjected to Western blot analysis with antibodies against indicated proteins. Abbreviations: STAT5-PY, phospho-Y694-STAT5. (**B**) 32D/ITD cells were left untreated or treated for 6 h with 100 nM AC-220, 10 μM Pimozide, or 1 μM AZD1208, as indicated, and analyzed. (**C**) 32D/ITD cells were cultured for 48 h with or without 1 μM GDC-0941 (GDC), 3 μM MK-2206 (MK), or 1 μM AZD1208 (AZD), as indicated, and viable cell numbers were counted after trypan blue staining. The means of relative cell numbers, expressed as percentages of control cells without inhibitors, from triplicate measurements are shown with error bars indicating standard errors. (**D**) 32D/ITD cells were treated for 48 h with or without 3 μM GDC-0941 (GDC), 3 μM MK-2206 (MK), or 1 μM AZD1208 (AZD), as indicated, and analyzed for the cellular DNA content by flow cytometry. Percentages of apoptotic cells with sub-G1 DNA content are indicated. (**E**) 32D/ITD cells were treated for 48 h with or without 3 μM GDC-0941 (GDC) or 1 μM AZD1208 (AZD), as indicated, and analyzed for activation of Bax or Bak, as indicated, by flow cytometry. (**F**) 32D/ITD cells were treated for 48 h with or without 1 μM GDC-0941 (GDC) or 1 μM AZD1208 (AZD), as indicated, and analyzed for activation of Caspase-3 by flow cytometry. (**G**) 32D/TKD cells transduced with STAT5A1*6 or vector control cells (Cont.) were lysed and subjected to Western blot analysis with antibodies against indicated proteins. (**H**) 32D/TKD cells transduced with STAT5A1*6 or vector control cells (Cont.) were cultured for 48 h with or without 3 μM GDC-0941 (GDC) or 1 μM AZD1208 (AZD), as indicated, and analyzed. (**I**) MV4-11 cells were treated for 16 h with 50 nM AC-220, 10 μM pimozide, or 1 μM AZD1208, as indicated, and analyzed. HSP90 was used for a loading control. (**J**) MV4-11 cells were cultured for 48 h with indicated concentrations of GDC-0941 and AZD1208, and viable cell numbers were measured by using the Cell counting Kit-8. The means of relative viable cell numbers obtained from triplicate measurements are plotted with error bars indicating standard errors. Combination index (CI) values obtained by the method of Chou and Talalay are also indicated. (**K**) MV4-11 cells were cultured for 48 h with or without 1 μM GDC-0941 (GDC) or 3 μM AZD1208 (AZD), as indicated, and analyzed. (**L**) MV4-11 cells were cultured for 48 h with or without 1 μM GDC-0941 (GDC) or 1 μM AZD1208 (AZD), as indicated, for analysis for activation of Caspase-3. Percentages of cells with cleaved Caspase-3 are indicated. (**M**) MV4-11 cells were cultured for 48 h with both 2 μM GDC-0941 and 3 μM AZD1208 (GDC/AZD) with or without 100 μM Boc-d-FMK, as indicated, and analyzed.

It has been reported that FLT3-ITD increases expression levels of Pim kinases mainly through STAT5 activation [3, 28, 29]. In accordance with this, 32D/ITD expressed Pim-1 at a higher level than 32D/TKD cells, which correlated with STAT5 activation in these cells (Figure 1A), whereas we could not clearly detect the Pim-2 expression in these cells (negative data not shown). Furthermore, abrogation of STAT5 activation by the FLT3 inhibitor AC-220 drastically reduced expression levels of the two isoforms of Pim-1 in 32D/ITD cells, while a modest downregulation of STAT5 activation by its inhibitor pimozide modestly reduced the Pim-1 expression (Figure 1B). On the other hand, the pan-Pim kinase inhibitor AZD1208 increased the expression level of Pim-1 in 32D/ITD cells most likely through stabilization in accordance with previous reports (Figure 1B) [32, 33]. Thus, it was confirmed that Pim-1 was inducibly expressed by FLT3-ITD through the STAT5 pathway in the 32Dcl3 model cell system we have employed.

To examine the possibility that Pim kinases mediate the resistance of FLT3-ITD-expressing cells to the PI3K/Akt inhibitors, we examined the effects of AZD1208 on 32D/ITD cells. As shown in Figure 1C, treatment with AZD1208 at 1 μM for 48 h did not show any significant effect on cell viability of 32D/ITD. Nevertheless, AZD1208 showed significant effects to reduce viability of cells co-treated with the PI3K inhibitor GDC-0941 or the Akt inhibitor MK-2206. Furthermore, AZD1208 drastically induced apoptosis in 32D/ITD cells when added in combination with GDC-0941 or MK-2206 as judged by increases in cells with sub-G1 cellular DNA content, a hallmark for apoptotic cells, while treatment with AZD1208 alone did not significantly induce apoptosis and GDC-0941 or MK-2206 induced apoptosis only modestly under these conditions (Figure 1D). The other pan-Pim kinase inhibitor PIM447 similarly induced apoptosis synergistically with GDC-0941 or MK-2206 in 32D/ITD cells (Supplementary Figure 1A). Consistent with these observations, the combined treatment of 32D/ITD cells with AZD1208 and GDC-0941 remarkably activated Bax as well as Bak, whereas AZD1208 alone failed to induce activation of these pro-apoptotic Bcl-2 family members involved in activation of the intrinsic apoptotic pathway and GDC-0941 showed only modest effects (Figure 1E). Furthermore, the executioner caspase Caspase-3 was cleaved and thus activated in the majority of 32D/ITD cells treated with both AZD1208 and GDC-0941, while it was activated in only small portions of cells treated with either of these inhibitors alone (Figure 1F).

To confirm that the Pim kinases mediate the resistance downstream of STAT5 in 32D/ITD cells, we next examined 32D/TKD cells expressing a constitutively activated STAT5 mutant, STAT5A1*6 [27]. As expected, Pim-1 was expressed at a higher level in STAT5A1*6-expressing 32D/TKD cells than in the vector control cells (Figure 1G). As shown in Figure 1H, inhibition of PI3K failed to induce apoptosis significantly in STAT5A1*6-expressing 32D/TKD cells in contrast to the vector control cells, which is consistent with our previous report [21]. However, inhibition of Pim-1 by AZD1208 abrogated the resistance to GDC-0941 endowed by STAT5A1*6 resulting in a remarkable induction of apoptosis with GDC-0941 in these cells. Together, these data suggest that the resistance of 32D/ITD cells to inhibitors of the PI3K/Akt pathway may be mainly mediated by induction of Pim-1 expression downstream of STAT5 activation in these cells.

We next examined the combined effect of AZD1208 and GDC-0941 on the FLT3-ITD-positive human AML cell line MV4-11. MV4-11 cells expressed the three isoforms of Pim-2, expression levels of which were reduced by AC-220 and pimozide in correlation with down regulation of STAT5 and were increased by AZD1208 similarly with the expression levels of Pim-1 in 32D/ITD cells (Figure 1B, 1I). On the other hand, Pim-1 was barely detectable in MV4-11 cells by immunoblot analysis (negative data not shown). As shown in Figure 1J, AZD1208 and GDC-0941 synergistically reduced viable cell numbers of MV4-11, as judged by combination index (CI) values obtained by the method of Chuo and Talalay [34] being less than 1 at all the concentrations examined. Although inhibiting cell proliferation, AZD1208 failed to induce apoptosis significantly in MV4-11 cells (Figure 1K). However, AZD1208 distinctively enhanced apoptosis as well as activation of Caspase-3 induced by GDC-0941 in MV4-11 cells (Figure 1K, 1L), which was inhibited by co-treatment with the pan-caspase inhibitor Boc-d-FMK (Figure 1M). Consistent with this, PIM447 remarkably enhanced apoptosis induced by GDC-0941 in MV4-11 cells (Supplementary Figure 1B). Together, these results suggest that inhibition of Pim kinases and the PI3K/Akt pathways synergistically reduced viability of 32D/ITD and MV4-11 cells at least partly through induction of caspase-dependent apoptosis mediated through the intrinsic pathway, which supports our hypothesis that Pim kinases mediate the resistance to the PI3K/Akt inhibitors downstream of STAT5 activation in FLT3-ITD-expressing cells.

### Inhibition of Pim kinases and PI3K cooperatively down regulates the mTORC1/Mcl-1 pathway

**Figure 2:**
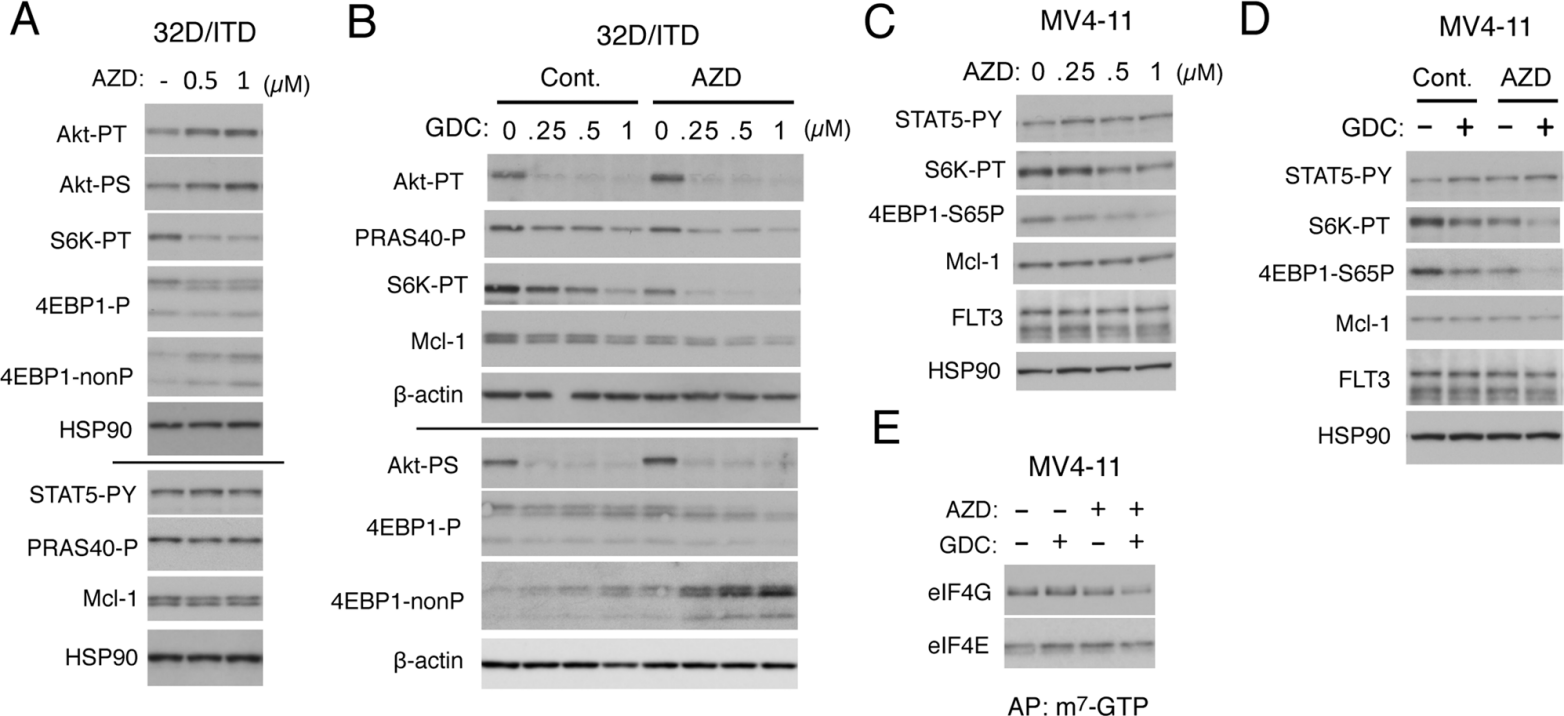
Inhibition of Pim kinases and PI3K cooperatively down regulates the mTORC1/Mcl-1 pathway in FLT3-ITD-expressing cells (**A**) 32D/ITD cells were cultured for 6 h with indicated concentrations of AZD1208 (AZD) and lysed. Cell lysates were run on duplicate gels and subjected to Western blot analysis with antibodies against indicated proteins. The results obtained from duplicate gels are shown above or below a thin horizontal line. Abbreviations: Akt-PT, phospho-T308-Akt; Akt-PS, phospho-S473-Akt; S6K-PT, phospho-T389-p70S6 kinase; 4EBP1-P, phospho-T37/46-4EBP1; 4EBP1-nonP, non-phospho-T46-4EBP1; STAT5-PY, phospho-Y694-STAT5; PRAS40-P, phospho-T246-PRAS40. (**B**) 32D/ITD cells were cultured for 6 h with indicated concentrations of GDC-0941 (GDC) in the presence or absence of 1 μM AZD1208 (AZD), as indicated, and analyzed as in A. (**C**) MV4-11 cells were cultured for 18 h with indicated concentrations of AZD1208 (AZD) and analyzed. 4EBP1-S65P: phospho-S65-4EBP1. (**D**) MV4-11 cells were cultured for 18 h with or without 0.25 μM GDC-0941 (GDC) and 1 μM AZD1208 (AZD), as indicated, and analyzed. (**E**) MV4-11 cells were cultured for 6 h with 0.25 μM GDC-0941 (GDC) or 1 μM AZD1208 (AZD), as indicated, and subjected to the cap-binding assay to analyze the eIF4E/eIF4G complex formation. Proteins affinity purified (AP) with m^7^-GTP-sepharose (m^7^-GTP) were subjected to Western blot analysis.

We have previously revealed that FLT3-ITD confers resistance to the PI3K/Akt pathway inhibition by protecting the downstream mTORC1/Mcl-1 pathway through robust activation of STAT5 [21]. Thus, we examined the possibility that Pim kinases may be involved in activation of the mTORC1 pathway in FLT3-ITD-expressing cells. As shown in Figure 2A, the pan-Pim kinase inhibitor AZD1208 did not show any significant effect on tyrosine phosphorylation of the FLT3-ITD substrate STAT5. Intriguingly, in repeated experiments, AZD1208 enhanced, though modestly, activation-specific phosphorylation of Akt on T308 and S473, which are target sites for PDK1 and mTORC2, respectively (Figure 2A, 2B, and data not shown). On the other hand, AZD1208 dose-dependently inhibited mTORC1 as judged by dephosphorylation of its direct substrates p70S6K and 4EBP1. Under these conditions, we did not observe any effect of AZD1208 on the level of Mcl-1 expression as well as on phosphorylation of PRAS40, which is a substrate of both Akt and mTORC1 and involved in regulation of the mTORC1 activity [35] (Figure 2A). These results suggest that Pim kinases may play a negative or positive role in activation of Akt or its downstream signaling component mTORC1, respectively, in FLT3-ITD-expressing cells.

We next examined the combined effect of the PI3K inhibitor GDC-0941 and AZD1208 on the PI3K/Akt/mTORC1/Mcl-1 pathway in 32D/ITD cells. In accordance with our previous report [21], GDC-0941 alone very efficiently inhibited phosphorylation of Akt on T308 and S473 but only moderately downregulated the mTORC1 pathway, as judged by phosphorylation of p70S6K and 4EBP1, in a dose-dependent manner (Figure 2B). However, when combined with AZD1208, GDC-0941 prominently inhibited the mTORC1 pathway at low concentrations and distinctively decreased the expression level of Mc1-1 and phosphorylation of PRAS40 in these cells. AZD1208 showed a modest inhibitory effect on the mTORC1 pathway also in MV4-11 cells without affecting STAT5 phosphorylation and prominently enhanced the inhibitory effect of GDC-0941 on this pathway to reduce the Mcl-1 expression level (Figure 2C, 2D). The other pan-Pim kinase inhibitor PIM447 also downregulated the mTORC1/Mcl-1 pathway cooperatively with GDC-0941 in 32D/ITD and MV4-11 cells in similar manners with AZD1208 (Supplementary Figure 2A–2D). We next performed the pull-down assays using m^7^-GTP beads to evaluate the effect of AZD1208 and GDC-0941 on formation of the eIF4E/eIF4G complex, which enhances the cap-dependent translation of mRNAs having lengthy, G+C-rich, highly structured 5’-UTRs, such as Mcl-1 mRNA [15]. As shown in Figure 2E, under the conditions where GDC-0941 or AZD1208 alone did not significantly reduce the amount of eIF4G pulled down with eIF4E bound to m^7^-GTP in MV4-11 cells, the combination of these inhibitors distinctively inhibited the eIF4E/eIF4G complex formation. Together, these results suggest that Pim kinases may protect the mTORC1 pathway to sustain the cap-dependent translation dependent on the eIF4E/eIF4G complex to maintain the Mcl-1 expression level in FLT3-ITD-expressing cells.

### Apoptosis induced cooperatively by inhibitors for PI3K and Pim kinases is mediated through downregulation of mTOR

**Figure 3:**
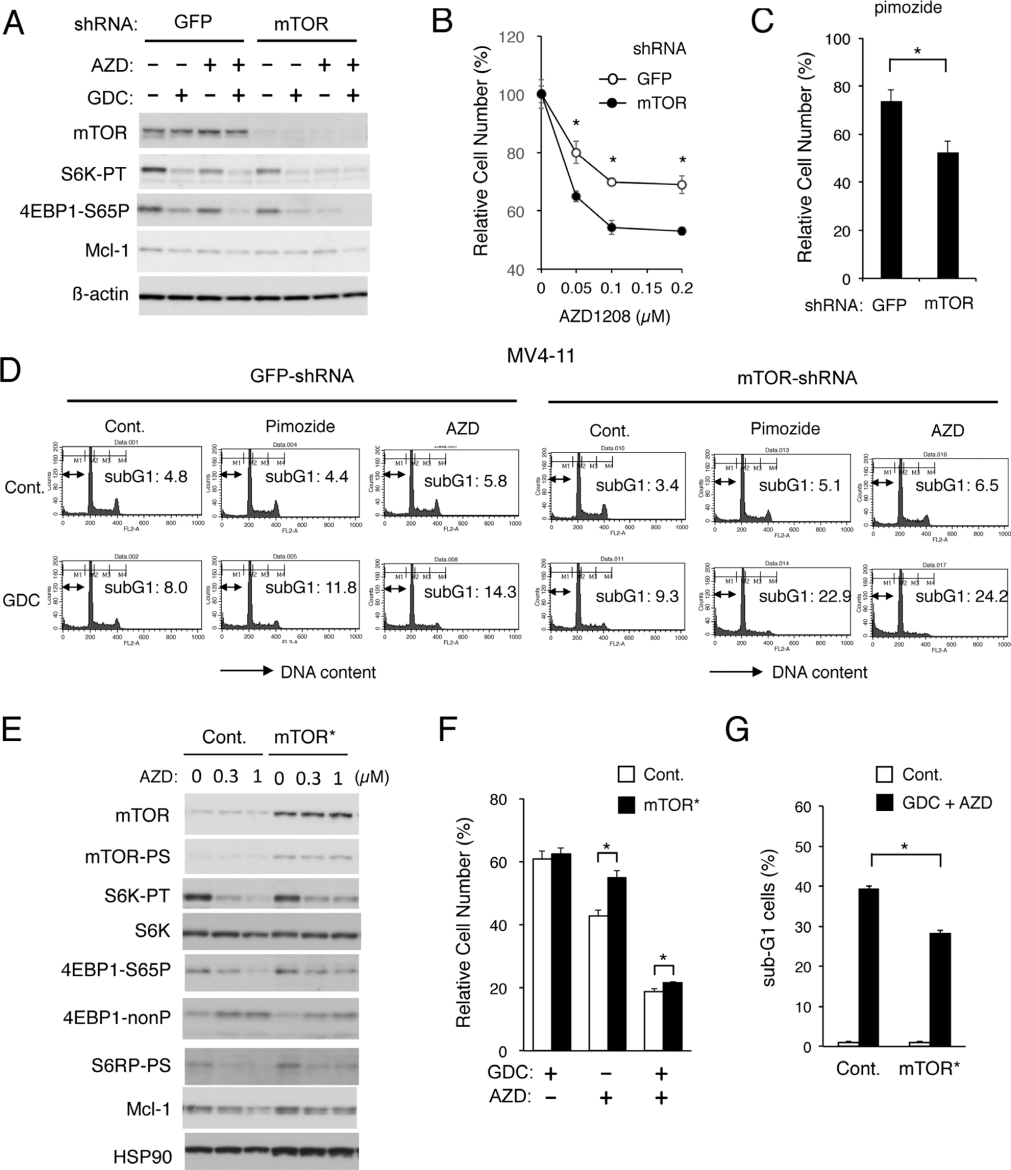
Apoptosis induced cooperatively by inhibitors for PI3K and Pim kinases is mediated through downregulation of mTOR (**A**) MV4-11 cells transduced with shRNA targeting for GFP or mTOR, as indicated, were lysed and subjected to Western blot analysis with antibodies against indicated proteins. Abbreviations: S6K-TP, phospho-T389-p70S6 kinase; 4EBP1-S65P, phospho-S65-4EBP1. (**B**, **C**) MV4-11/GFP-shRNA or MV4-11/mTOR-shRNA cells, as indicated, were cultured for 48 h with indicated concentrations of AZD1208 (B) or 2.5 μM pimozide (C). The means of relative viable cell numbers, expressed as percentages of control cells without inhibitors, from triplicate measurements are plotted with error bars indicating standard errors. The asterisks indicate statistically significant differences determined by Student’s *t*-test (^*^P < 0.05). (**D**) MV4-11/GFP-shRNA or MV4-11/mTOR-shRNA cells, as indicated, were treated for 48 h with indicated concentrations of GDC-0941 (GDC), pimozide, or AZD1208 (AZD) and analyzed for the cellular DNA content by flow cytometry. Percentages of apoptotic cells with sub-G1 DNA content are indicated. (**E**) 32D/ITD cells transduced with the activated mTOR mutant mTOR-E2419K (mTOR^*^) or vector control cells (Cont.), as indicated, were treated for 6 h with indicated concentrations AZD1208 (AZD) and subjected to Western blot analysis with antibodies against indicated proteins. Abbreviations: mTOR-PS, phospho-S2481-mTOR; S6K-PT, phospho-T389-p70S6 kinase; S6K, p70S6 kinase; 4EBP1-nonP, non-phospho-T46-4EBP1; S6RP-PS, phosphor-S240/244-S6RP. (**F**) 32D/ITD cells expressing mTOR-E2419K (mTOR^*^) or vector control cells (Cont.) were cultured for 48 h with 0.5 μM GDC-0941 (GDC) or 0.5 μM AZD1208 (AZD), as indicated, in triplicate. The means of relative viable cell numbers, expressed as percentages of control cells without inhibitors, from triplicate measurements are shown with error bars indicating standard errors. The asterisks indicate statistically significant differences determined by Student’s *t*-test (^*^P < 0.05). (**G**) 32D/ITD cells expressing mTOR-E2419K (mTOR^*^) or vector control cells (Cont.) were cultured for 48 h with or without 3 μM GDC-0941 and 2 μM AZD, as indicated, in triplicate, and analyzed for the cellular DNA content by flow cytometry. The means of percentages of apoptotic cells with sub-G1 DNA content are shown with error bars indicating standard errors. The asterisks indicate statistically significant differences determined by Student’s *t*-test (^*^P < 0.05).

To explore the involvement of Pim kinases in modulation of the mTORC1/Mcl-1 pathway regulating proliferation and survival of FLT3-ITD-expressing cells, we next examined MV4-11 cells infected with mTOR_1 shRNA to knock down the mTOR expression. The expression level of mTOR in these cells was drastically reduced as compared with that in cells infected with the control pLKO.1 puro GFP siRNA, whereas levels of phosphorylation of p70S6K and 4EBP1 as well as an expression level of Mcl-1 in these cells were only slightly reduced as compared with control cells cultured under optimal conditions (Figure 3A). However, AZD1208 and, to the lesser extent, GDC-0941 reduced phosphorylation of p70S6K and 4EBP1 more distinctively in mTOR-knocked down cells than in control cells, with the corresponding decline in Mcl-1 expression levels observed when treated with both AZD1208 and GDC-0941. In accordance with this, AZD1208 as well as the STAT5 inhibitor pimozide reduced the viable cell number of mTOR-knocked down cells more significantly than that of control cells (Figure 3B, 3C). Furthermore, apoptosis induced by the combined treatment with GDC-0941 and pimozide or AZD1208 was much more prominently observed in mTOR-knocked down MV4-11 cells than in control cells, while these inhibitors when used alone induced apoptosis only modestly and comparably in these cells (Figure 3D).

Next, we examined 32D/ITD cells expressing a constitutively-activated mutant of mTOR, mTOR-E2419K [36]. As shown in Figure 3E, these cells expressed the activated form of mTOR phosphorylated on S2481 as well as total mTOR at a much higher level than vector control cells. As compared with vector control cells, 32D/ITD cells expressing mTOR-E2419K showed resistance to the inhibitory effect of AZD1208 on the mTORC1/Mcl-1 pathway (Figure 3E). In accordance with this, AZD1208 reduced the viable cell number of 32D/ITD cells expressing mTOR-E2419K less significantly than that of control cells (Figure 3F). Furthermore, the combined treatment with GDC-0941 and AZD1208 induced apoptosis less significantly in 32D/ITD cells expressing mTOR-E2419K than in control cells (Figure 3G and Supplementary Figure 3). These results support the idea that upregulation of the mTORC1/Mcl-1 pathway by Pim kinases may play an important role in acquisition of the resistance to the PI3K/Akt inhibitors by FLT3-ITD-expressing cells.

### Mcl-1 mediates acquisition of the resistance to PI3K inhibition downstream of Pim kinases in FLT3-ITD-expressing cells

**Figure 4:**
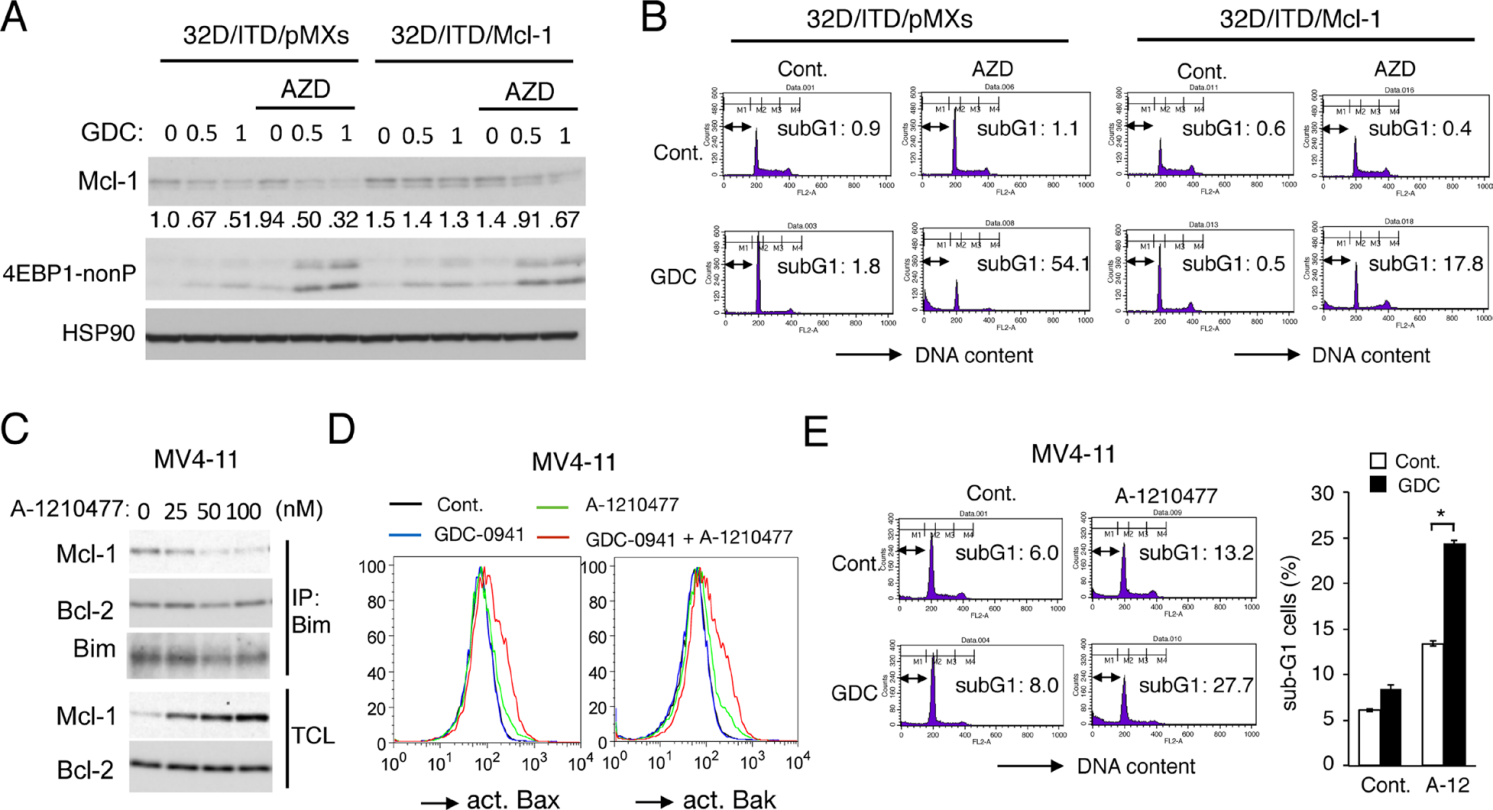
Mcl-1 mediates the acquisition of resistance to PI3K inhibition downstream of Pim kinases in FLT3-ITD-expressing cells (**A**) 32D/ITD cells transduced with Mcl-1 (32D/ITD/Mcl-1) or vector control cells (32D/ITD/pMXs), as indicated, were treated for 6 h with indicated concentrations of GDC-0941 (GDC) with or without 1 μM AZD1208 (AZD), as indicated, and subjected to Western blot analysis with antibodies against indicated proteins. 4EBP1-nonP: non-phospho-T46-4EBP1. Relative expression levels of Mcl-1 were determined by densitometric analysis and are shown below the panel. (**B**) 32D/ITD/pMXs or 32D/ITD/Mcl-1 cells were treated for 48 h with 3 μM GDC-0941 (GDC) or 1 μM AZD1208 (AZD), as indicated, and analyzed for the cellular DNA content by flow cytometry. Percentages of apoptotic cells with sub-G1 DNA content are indicated. (**C**) MV4-11 cells were cultured for 6 h in serum free medium with indicated concentrations of A-1210477. Total cell lysates (TCL) and immunoprecipitates (IP) with anti-Bim antibody were subjected to immunoblot analysis using indicated antibodies. (**D**) MV4-11 cells were treated in serum free medium for 24 h with 1 μM GDC-0941 or 50 nM A-1210477, as indicated, and subjected to flow cytometric analyses for activation of Bax or Bak, using activation-specific antibodies. (**E**, **F**) MV4-11 cells were cultured in serum free medium for 24 h with 0.5 μM GDC-0941 or 25 nM A-1210477, as indicated, and analyzed for the cellular DNA content by flow cytometry. Percentages of apoptotic cells with sub-G1 DNA content are indicated in representative data, and the means of triplicate measurements are plotted with error bars indicating standard errors. The asterisks indicate statistically significant differences determined by Student’s *t*-test (^*^P < 0.05).

To confirm that Pim kinases may mediate protection of the mTORC1/Mcl-1 pathway to confer the resistance to PI3K/Akt pathway inhibitors in FLT3-ITD-expressing cells, we next examined 32D/ITD cells overexpressing Mcl-1. As shown in Figure 4A, the 4EBP1 phosphorylation was efficiently inhibited by the combined treatment with GDC-0941 and AZD1208 in 32D/ITD cells overexpressing Mcl-1 as well as in vector control cells. However, the Mcl-1 expression level in cells transduced with the Mcl-1 expression vector was less significantly reduced by the combined treatment as compared with that in vector control cells. This is expected because only the expression of endogenous Mcl-1 should be significantly reduced by inhibition of the cap-dependent translation dependent on the eIF4E/eIF4G complex. As shown in Figure 4B, the combined treatment with GDC-0941 and AZD1208 induced apoptosis prominently and synergistically in vector control cells, which, however, was significantly reduced in Mcl-1-overexpressing cells. These results strongly suggest that the protection of the mTORC1/Mcl-1 pathway by the Pim kinases may play a role in acquisition of the resistance to PI3K inhibition.

To further confirm the significance of Mcl-1 on the resistance of FLT3-ITD-positive AML cells to inhibition of the PI3K/Akt pathway, we next examined effects of the Mcl-1-specific BH3-mimetic A-1210477 [37] on MV4-11 cells. First, we confirmed that A-1210477, at as low as 25 nM, reduced binding of Mcl-1 with the BH3-only pro-apoptotic protein Bim, while dose-dependently increasing the expression level of Mcl-1 as reported previously [37], which is expected from binding of A-1210477 to the Mcl-1 BH3 binding groove required for ubiquitination and degradation of Mcl-1 through the ubiquitin/proteasome system [17] (Figure 4C). Next, we examined the effect of A-1210477 on activation of the pro-apoptotic Bcl-2 family members Bax and Bak required for activation of the intrinsic mitochondria-mediated apoptotic pathway. As shown in Figure 4D, treatment of MV4-11 cells with GDC-0941 alone apparently activated neither Bax nor Bak, while A-1210477 only slightly induced activation of them. In contrast, Bax as well as Bak was distinctively activated by the combined treatment with these inhibitors. Consistent with this, GDC-0941 showed a more remarkable effect to increase apoptosis of MV4-11 cells in the presence of A-1210477 than in its absence (Figure 4E, 4F). Thus, inhibition of Mcl-1 enhanced apoptosis induced by GDC-0941 in MV4-11 cells in a similar manner with inhibition of Pim kinases. Together, these results support the idea that Pim kinases may mediate the resistance to the PI3K/Akt pathway inhibitors in FLT3-ITD-expressing cells at least partly by sustaining the Mcl-1 expression downstream of mTORC1.

### Overexpression of Pim-1 upregulates the mTORC1 pathway and confers the resistance to PI3K/Akt inhibitors on FLT3-TKD-expressing cells

**Figure 5:**
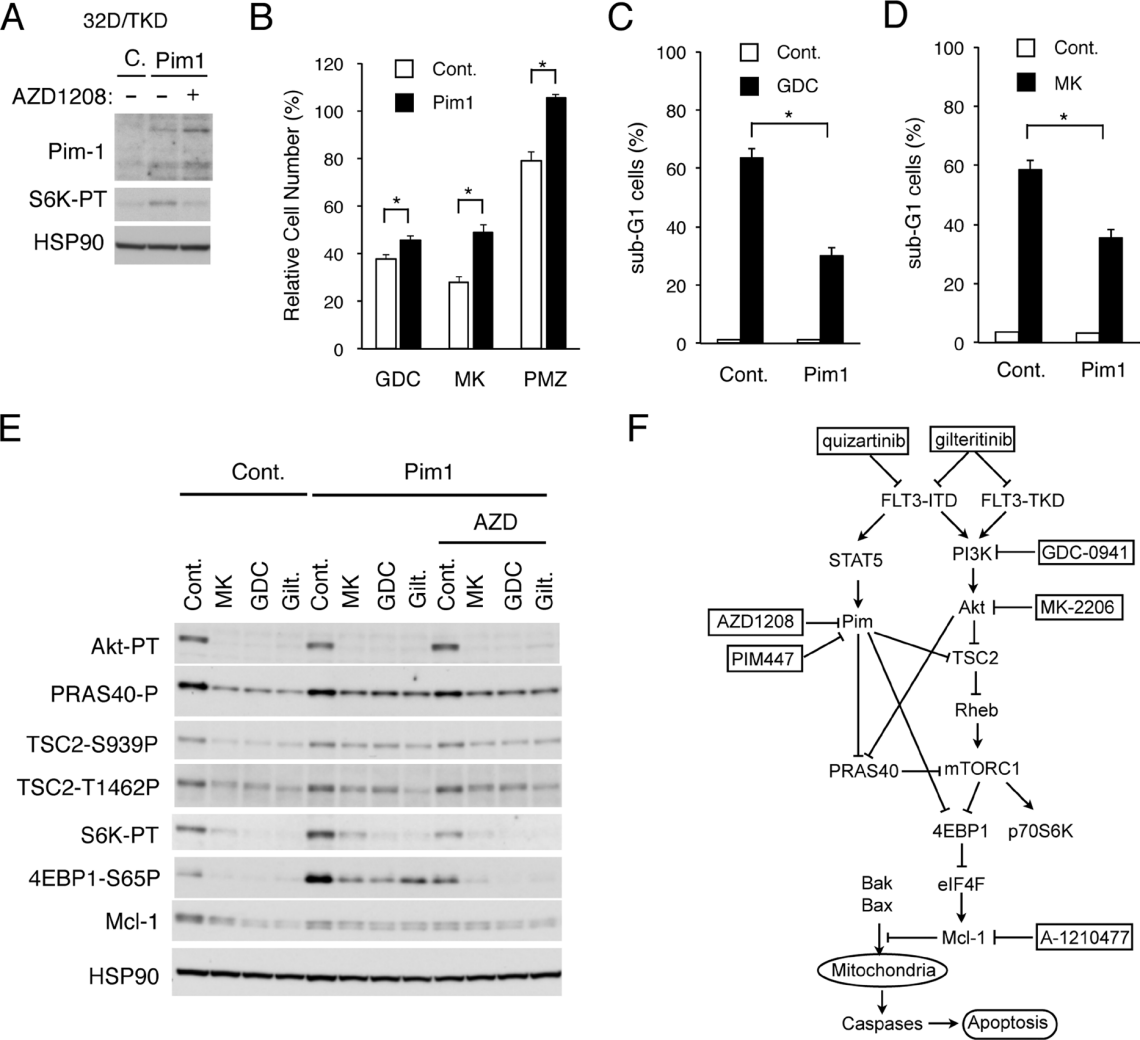
Overexpression of Pim-1 upregulates the mTORC1 pathway and confers the resistance to PI3K/Akt inhibitors on FLT3-TKD-expressing cells (**A**) 32D/TKD cells overexpressing Pim-1 (Pim1) or vector control cells (C) were treated with or without 1 μM AZD1208 for 6 h, as indicated, and lysed for Western blot analysis with antibodies against indicated proteins. S6K-TP: phospho-T389-p70S6 kinase. (**B**) 32D/TKD cells overexpressing Pim-1 (Pim1) or vector control cells (Cont.) were cultured for 48 h with 0.4 μM GDC-0941 (GDC), 0.2 μM MK-2206 (MK) or 2 μM Pimozide (PMZ), as indicated. The means of relative cell numbers, expressed as percentages of control cells without inhibitors, from triplicate measurements are shown with error bars indicating standard errors. The asterisks indicate statistically significant differences determined by Student’s *t*-test (^*^P < 0.05). (**C**, **D**) 32D/TKD cells overexpressing Pim-1 (Pim1) or vector control cells (Cont.) were cultured for 48 h with 3 μM GDC-0941 (GDC) or 2 μM MK-2206 (MK), as indicated, and analyzed for the cellular DNA content by flow cytometry. The means of percentages of apoptotic cells with sub-G1 DNA content are shown with error bars indicating standard errors. The asterisks indicate statistically significant differences determined by Student’s *t*-test (^*^P < 0.05). (**E**) 32D/TKD cells overexpressing Pim-1 (Pim1) or vector control cells (Cont.) were cultured for 6 h with 1 μM MK-2206 (MK), 1 μM GDC-0941 (GDC), 50 nM gilteritinib (Gilt.), or 1 μM AZD1208 (AZD), as indicated. Cells were lysed and subjected to Western blot analysis with antibodies against indicated proteins. Abbreviations: Akt-PT, phospho-T308-Akt; PRAS40-P, phospho-T246-PRAS40; TSC2-S939-P, phospho-S939-TSC2; TSC2-T1462P, phospho-T1462-TSC2; mTOR-PS, phospho-S2481-mTOR; S6K-PT, phospho-T389-p70S6 kinase; 4EBP1-S65P, phospho-S65-4EBP1. (**F**) A schematic model of intracellular signaling mechanisms involving enhancement of the mTORC1/Mcl-1 pathway by Pim kinases expressed through STAT5 activation in regulation of survival and proliferation of FLT3-ITD-positive AML cells in response to the PI3K/Akt pathway inhibitors.

To confirm further the involvement of Pim kinases in acquisition of resistance to the PI3K/Akt pathway inhibitors by FLT3-ITD-expressing cells, we examined 32D/TKD cells overexpressed Pim-1 (Figure 5A). As compared with vector control cells, phosphorylation of the mTORC1 substrate p70S6K was increased in these cells and was partially inhibited by the Pim inhibitor AZD1208 as expected (Figure 5A). As shown in Figure 5B, GDC-0941 or MK-2206 reduced the viable cell number of Pim-1-overexpressing 32D/TKD cells less significantly than that of vector control cells. Furthermore, the STAT5 inhibitor pimozide reduced the viable cell number of vector control cells but not that of Pim-1-overexpressing cells. In accordance with this, apoptosis induced by GDC-0941 or MK-2206 in 32D/TKD cells was significantly reduced by overexpression of Pim-1 (Figure 5C and 5D). As shown in Figure 5E, phosphorylation of Akt on T308 and its inhibition by GDC-0941, MK-2206, or the FLT3 inhibitor gilteritinib, which inhibits FLT3-TKD as well as FLT3-ITD unlike AC-220 [38], were observed similarly in Pim-1-overexpressing 32D/TKD cells and in vector control cells. However, the mTORC1 activity, as determined by phosphorylation of its target p70S6K in the Pim-1-overexpressing cells was distinctively increased as compared with that in vector control cells (Figure 5A, 5E). Moreover, phosphorylation of 4EBP1 on S65, which is also a target site for mTORC1, was drastically increased in Pim-1-overexpressing 32D/TKD cells and showed a partial resistance to the inhibitory effect of GDC-0941, MK-2206, or gilteritinib. As expected, the Pim kinase inhibitor AZD1208 decreased the phosphorylation of p70S6K and 4EBP1 in Pim-1-overexpressing 32D/TKD cells and sensitized that of 4EBP1 to the inhibitory effect of GDC-0941, MK-2206, or gilteritinib. Although the Mcl-1 expression in Pim-1-overexpressing cells was not increased as compared with that in control cells, it also showed the resistance to these inhibitors to be downregulated. Intriguingly, phosphorylation of TSC2 on S939 or T1462 and PRAS40 on T246, the target sites for Akt playing important roles in activation of the downstream mTORC1 pathway, was not increased distinctively but showed the resistant to inhibitory effects of GDC-0941, MK-2206, and gilteritinib in Pim-1-overexpressing cells as compared with that in control cells. Together, these data suggest that Pim-1 may increase activation of mTORC1 cooperatively with Akt by directly or indirectly enhancing phosphorylation of 4EBP1 on S65, TSC2 on S939 and T1462, and PRAS40 on T246 to confer on the mTORC1/Mcl-1 pathway the resistance to inhibition of the PI3K/Akt pathway in FLT3-ITD-expressing cells (Figure 5F).

### Inhibition of Pim kinases and PI3K cooperatively reduces Mcl-1 expression and viable cell numbers of some primary AML cells expressing FLT3-ITD

**Figure 6:**
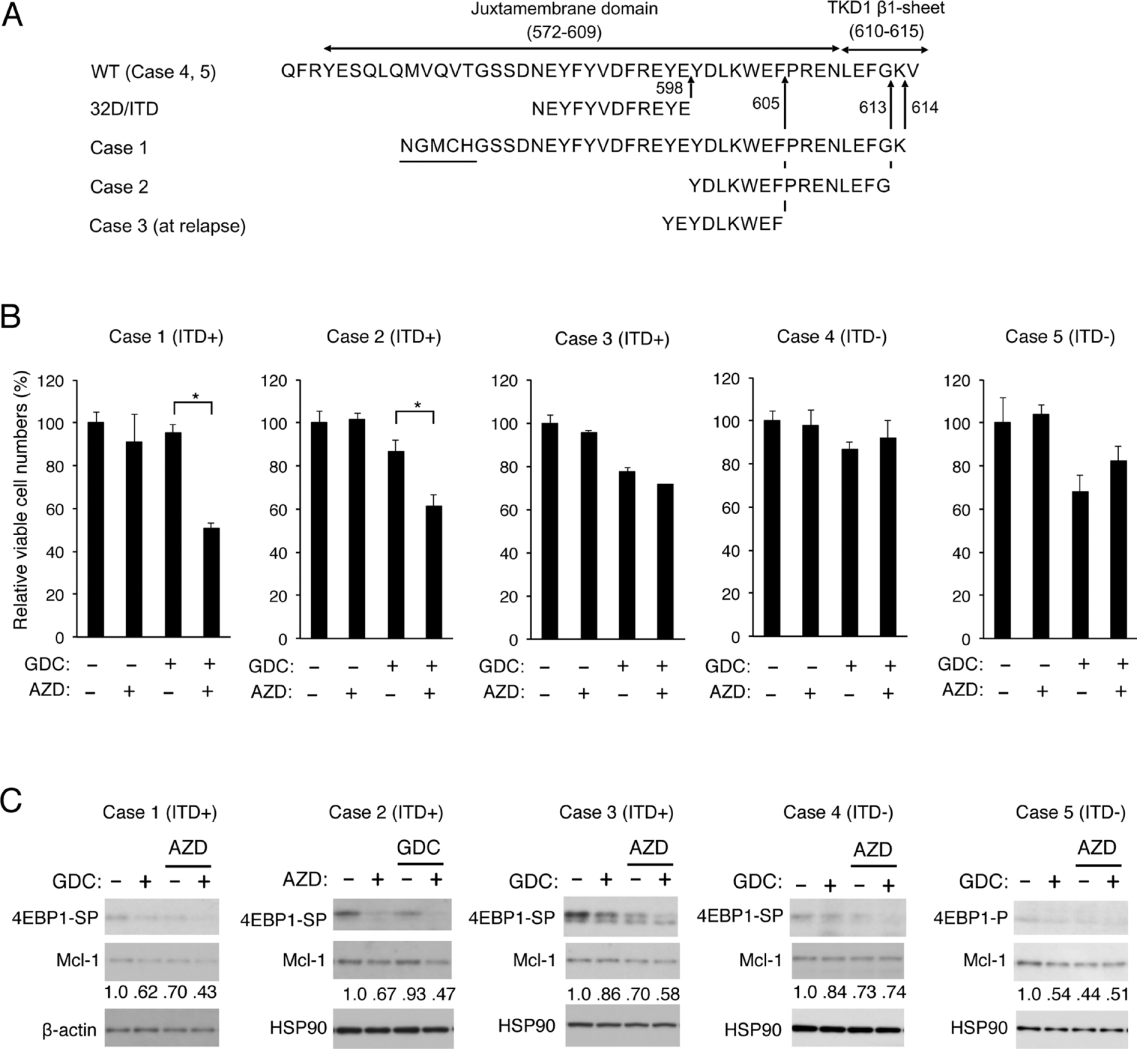
Inhibition of Pim kinases and PI3K cooperatively reduced Mcl-1 expression and viable cell numbers of some primary AML cells expressing FLT3-ITD (**A**) Partial amino acid sequences of wild-type FLT3 (WT) and inserted amino acid sequences of FLT3-ITD expressed in 32D/ITD or in primary AML cells from Cases 1, 2, and 3 are shown. The insertion sites are indicated by arrows and amino-acid numbers. Sequences unrelated to FLT3 found inserted in Case 1 are underlined. Case 1 is identical with that reported previously [21]. (**B**) Primary AML cells from FLT3-ITD-positive (Cases 1-3) or -negative (Case 4, 5) patients were cultured with or without GDC-0941 (GDC) and AZD1208 (AZD) at 0.25 μM (Case 1, 2) or 0.5 μM (Case 3-5) for 24 h (Case 2, 4, 5), 48 h (Case 3), or 72 h (Case 1). The means of relative cell numbers, expressed as percentages of control cells without inhibitors, from triplicate measurements are shown with error bars indicating standard errors. The asterisks indicate statistically significant differences determined by Student’s *t*-test (^*^P < 0.05). The sources and percentages of blasts are as follows: Case 1, the bone marrow (BM) at diagnosis, 95.4%; Case 2, the peripheral blood (PB) at diagnosis, 89%, Case 3, PB at relapse, 97%; Case 4, PB at diagnosis, 94%; Case 5, BM at diagnosis, 82.6%. (**C**) Primary AML cells from FLT3-ITD-positive (Cases 1-3) or -negative (Case 4, 5) patients were cultured with or without 0.1 μM (Case 1) or 0.2 μM (Case 2-5) GDC-0941 (GDC) and 0.5 μM (Case 1) or 0.3 μM (Case 2-5) AZD1208 (AZD), as indicated, for 8 h. Cells were lysed and subjected to Western blot analysis with antibodies against indicated proteins. Abbreviations: 4EBP1-SP, phospho-S65-4EBP1; 4EBP1-P, phospho-T37/46-4EBP1. Relative expression levels of Mcl-1 were determined by densitometric analysis and are shown below the panels.

We finally examined the combined effects of inhibition of PI3K and Pim kinases in primary leukemic cells from 5 AML patients with or without FLT3-ITD (Figure 6A). Two patients were found to have FLT3-ITD at diagnosis with their insertion sites in the tyrosine kinase domain (Case 1, 2), while FLT3-ITD inserted within the juxtamembrane domain was newly found at relapse in one patient (Case 3). Two samples from patients with AML at diagnosis without FLT3-ITD were also analyzed for comparison (Case 4, 5). Under the conditions where treatment with AZD1208 or GDC-0941 alone showed marginal effects, the effect of GDC-0941 to decrease the viable cell number was more prominently observed in the presence of AZD1208 than in its absence in 2 primary samples from FLT3-ITD-positive AML patients at diagnosis (Figure 6B; Case 1, 2), but not significantly in a sample from FLT3-ITD-positive AML patient at relapse (Case 3) or not at all in 2 samples without FLT3-ITD (Case 4, 5). We next examined the effects of GDC-0941 and AZD1208 on the mTORC1/4EBP1/Mcl-1 pathway in these primary leukemic cells by immunoblot analysis. In accordance with the data obtained with cell lines, AZD1208 as well as GDC-0941 showed inhibitory effects on phosphorylation of 4EBP1 to various extents in all the samples we could examine, including those from patients with AML without FLT3-ITD (Figure 6C). Importantly, the combined treatment with GDC-0941 and AZD1208 reduced the expression levels of Mcl-1 more distinctively as compared with treatment with either of these inhibitors alone only in FLT3-ITD-positive primary AML cells (Case 1-3), which correlated to some extent with the inhibitory effects on phosphorylation of 4EBP1 and on viable cell numbers. These results suggest that Pim kinases may protect the mTORC1/4EBP1/Mcl-1 pathway to confer resistance to the PI3K inhibitor on leukemic cells from at least some of patients with FLT3-ITD-positive AML.

## DISCUSSION

In the present study, we have extended our previous study [21] by revealing that Pim kinases are involved downstream of the robust STAT5 activation in acquisition of the resistance to PI3K/Akt inhibitors by protecting the mTORC1/Mcl-1 pathway in FLT3-ITD-expressing AML cells. This is because Pim-1 or Pim-2 was shown to be expressed downstream of STAT5 activation in 32D/ITD as well as 32D/TKD/STAT5A1*6 cells or MV4-11 cells, respectively, and inhibition of these kinases by the highly selective pan-Pim kinase inhibitor AZD1208 or PIM447 sensitized these cells to the PI3K inhibitor GDC-0941 as well as the Akt inhibitor MK-2206 for induction of apoptosis mediated through the intrinsic pathway by downregulating the mTORC1/Mcl-1 pathway. Thus, inhibition of Pim kinases induced very similar effects with inhibition of STAT5 by pimozide in FLT3-ITD-expressing cells we demonstrated in our previous study [21]. Furthermore, overexpression of Mcl-1 as well as that of Pim-1 conferred on 32D/TKD cells resistance to apoptosis induced through the intrinsic pathway by the PI3K/Akt inhibitors, while inhibition of Mcl-1 by its specific BH3-antagonist A-1210477 sensitized MV4-11 cells to GDC-0941 for induction of caspase-dependent apoptosis involving activation of pro-apoptotic Bcl2-family proteins Bax and Bak. As expected, overexpression of Pim-1 in 32D/TKD cells increased the activation level of the mTORC1/Mcl-1 pathway and partially protected it from downregulation by inhibition of the PI3K/Akt pathway as well as the FLT3-TKD kinase activity by its specific inhibitor gilteritinib. Finally, the combined inhibitory effects of AZD1208 and GDC-0941 on viable cell numbers and the mTORC1/Mcl-1 pathway were confirmed in primary leukemic cells from some patients with FLT3-ITD-positive AML, although the number of cases we could analyze was limited because of technical difficulties. Thus, the present study has revealed a critical role Pim kinases play in enhancement of the mTORC1 pathway downstream of the FLT3-ITD and suggests that these kinases are promising targets for the combined therapy with the PI3K/Akt pathway inhibitors against FLT3-ITD-positive AML associated with poor prognosis.

Increases in expression of the Pim kinases Pim-1 and Pim-2 by FLT3-ITD have been repeatedly reported in model hematopoietic cell lines, human AML cell lines, and primary AML cells [3, 28, 29]. Furthermore, Pim kinases have been shown to play important roles in FLT3-ITD-induced cell proliferation and survival. As for the difference between Pim-1 and Pim-2, however, conflicting results have been reported, because Adams M. et al. [39] demonstrated expression of kinase-dead Pim-2 mutant, but not that of Pim-1, led to a rapid decline of survival in IL-3-dependent Ba/F3 cells transformed by FLT3-ITD, while Kim DT et al. [28] showed that the kinase-dead Pim-1 accelerated cytotoxicity in response to FLT3 inhibition and inhibited colony growth of FLT3-ITD-transformed Ba/F3 cells. In the present study, Pim-1 or Pim-2 was confirmed to be expressed through STAT5 activation by FLT3-ITD in 32D/ITD or MV4-11 cells, respectively, and their inhibition by the pan-Pim kinase inhibitor AZD1208 showed indistinguishable effects on downstream signaling events and cell survival in combination with PI3K/Akt inhibitors in these cells. Consistent with this, a recent study has revealed that all three Pim kinases, including Pim-3, activated the indistinguishable downstream signaling cascades, protected FL5.12 cells from IL-3 withdrawal, and cooperated with Myc to induce myeloid leukemia in mice [40]. Thus, Pim-1 and Pim-2 may play indistinguishable and redundant roles in enhancement of the mTORC1 pathway in FLT3-ITD-expressing cells, which suggests that pan-Pim inhibitors should give more effective therapeutic effects against FLT3-ITD-positive AML than many Pim-1-selective inhibitors currently under development [23, 24]. In this regard, it should be noted that a planned clinical trial of AZD1208 in AML has been canceled due to highly variable pharmacokinetics and time-dependent decrease in exposure [41]. However, two pan-Pim kinase inhibitors, PIM447 (LGH477) and INCB53914, are currently in clinical trials and we have confirmed that PIM447 showed very similar effects with AZD1208 on FLT3-ITD expressing cells (Supplementary Figures 1 and 2).

It was previously reported that Pim kinase inhibitors, SGI-1776 and AR00459339, showed cytotoxic effects preferentially in AML cells with FLT3-ITD, thus implicating the Pim kinases as promising therapeutic targets for this type of AML with poor prognosis [42, 43]. Unlike AZD1208, however, SGI-1776 was later revealed to inhibit the FLT3 kinase activity directly in AML cells [30, 44]. AR00459339 also inhibited various signaling events downstream of FLT3-ITD, such as activation of STAT5 and Akt, which led the authors to speculate that Pim-1 may be involved in activation of these more “up-stream” signaling events [43]. However, in the present study, we demonstrate that the highly selective pan-Pim kinase inhibitor AZD1208 inhibited neither STAT5 nor Akt in AML cells expressing FLT3-ITD. Thus, the significance of Pim kinases as therapeutic targets in FLT3-ITD-positive AML remained elusive because of off target effects of these low-specific Pim kinase inhibitors. On the other hand, AZD1208 has previously been reported to inhibit proliferation of 5 of 14 AML cell lines, including MV4-11, with the inhibitory effect correlating with Pim-1 expression, STAT5 activation, and inhibitory effects of AZD1208 on mTORC1 activity [30]. Consistent with our results, however, AZD1208 alone did not induce apoptosis in these AML cell lines except for an exceptional cell line, Molm-16, carrying a Tyk2 fusion gene [45]. The authors also demonstrated that AZD1208 reduced colony formation of primary AML cells, which, however, was observed irrespective of the presence of FLT3-ITD. More recently, Meja K. et al. [46] reported that the pan Pim kinase inhibitor AZD1897 and the Akt inhibitor AZD5363 synergistically inhibited proliferation of various AML cell lines, which correlated with downregulation of the mTORC1 activity and Mcl-1 expression. Although apoptosis was induced by the combined treatment in some of the AML cell lines examined, those with FLT3-ITD, such as MV4-11, were not analyzed in their study. Furthermore, the authors did not find the significant correlation between the FLT3 mutations and the Pim-1 expression or the sensitivity to AZD1897 in primary AML samples. Therefore, the significance of Pim kinases as therapeutic targets in FLT3-ITD-driven AML cells has not adequately been addressed in these reports. In the present study, we have extended these previous studies by revealing that AZD1208 in combination with PI3K/Akt inhibitors not only inhibited proliferation but also induced apoptosis at least partly by reducing expression level of Mcl-1 downstream of mTORC1 pathway in FLT3-ITD-driven cells, including MV4-11, and that AZD1208 significantly enhanced the effects of GDC-0941 on viable cell numbers and Mcl-1 expression levels in some of primary AML cells expressing FLT3-ITD. Thus, the present study implies that Pim kinases would be promising targets in combination with the PI3K/Akt pathway for novel therapeutic strategies against AML with FLT3-ITD.

Pim kinases have been reported to upregulate the mTORC1 pathway through various mechanisms involving phosphorylation of TSC2 on S1798 [47], PRAS40 on T246 [48], and 4EBP1 on S65 [49] as well as inhibition of AMPK [50]. In the present study, we have demonstrated that overexpression or inhibition of Pim kinases upregulated or downregulated phosphorylation of 4EBP1 on S65, which might have been mediated directly by Pim kinases or indirectly by mTORC1. On the other hand, inhibition of Pim kinases downregulated phosphorylation of PRAS40 on T246 most remarkably in cells simultaneously treated with the PI3K inhibitor GDC-0941, while overexpression of Pim-1 partially prevented its dephosphorylation by treatment with the PI3K/Akt inhibitors as well as FLT3 inhibitor, thus suggesting that Pim kinases may be involved in phosphorylation of PRAS40 on T246 cooperatively with Akt. Although we could not directly examine phosphorylation of TSC2 on T1798 reported to be phosphorylated by Pim-2 in myeloma cells [47], that on S939 or T1462, known to be involved in downregulation of its GAP activity [35], became partly resistant to dephosphorylation by the PI3K/Akt inhibitors as well as the FLT3 inhibitor in 32D/TKD cells overexpressing Pim-1, which implies that Pim-1 may upregulate the mTORC1 pathway also at the level of TSC2 directly or indirectly through mechanisms not involving Akt. On the other hand, we failed to observe modulation of phosphorylation of AMPK by AZD1208 or by overexpression of Pim-1 under our experimental conditions (negative data not shown). Taken together with the observation that inhibition of Pim kinases rather increased Akt activation, these results imply that Pim kinases may upregulate the mTORC1/Mcl-1 pathway through several mechanisms involving TSC2, PRAS40, and 4EBP1 cooperatively with the PI3K/Akt pathway in FLT3-ITD-driven cells to prevent apoptosis (Figure 5F). Future studies, however, are required to explore possible mechanisms unrelated to the mTORC1/Mcl-1 pathway, such as inhibition of Bad or enhancement of CXCR4 signaling [16], through which Pim kinases may mediate to confer therapy resistance on FLT3-ITD-driven AML cells.

## MATERIALS AND METHODS

### Cells and reagents

Murine IL-3-dependent 32Dcl3 cells Ton.32D/FLT3-ITD (32D/ITD) or Ton.32D/FLT3-D835Y (32D/TKD) inducibly expressing FLT3-ITD or FLT3-D835Y, respectively, when cultured with doxycycline as well as 32D/TKD cells expressing the constitutively activated STAT5 mutant STAT5A1*6 (32D/TKD/STAT5A1*6) or 32D/ITD cells overexpressing Mcl-1 (32D/ITD/Mcl-1) and their vector control cells (32D/TKD/pMXs, 32D/ITD/pMXs) have been described previously [21, 51] and maintained in RPMI 1640 medium supplemented with 10% fetal calf serum (FCS) and 5 U/ml recombinant mIL-3 (PeproTech, Rocky Hill, NJ). Before analyses, these cells were cultured in medium containing doxycycline without IL-3 to proliferate dependent on FLT3-ITD or FLT3-TKD and independent of mIL-3. MV4-11 cells were purchased from ATCC and cultured in Iscove’s modified Dulbecco medium (IMDM) containing 10% FCS.

The PI3K inhibitor GDC-0941, the Pim inhibitor AZD1208, and the FLT3 inhibitor gilteritinib were purchased from Active Biochem (Kowloon, Hong Kong). The Akt inhibitor MK-2206 and the Pim inhibitor PIM447 were purchased from Selleckchem (Houston, TX). The FLT3 inhibitor AC-220 (quizartinib) was purchased from LC laboratories (Woburn, MA). The Mcl-1 inhibitor A-1210477 was purchased from Chemietek (Indianapolis, IN) Doxycycline, propidium iodide (PI), and antibody against β-actin (A1978) were purchased from Sigma Aldrich (St. Louis, MO). The STAT5 inhibitor pimozide (sc-203662) and antibodies against HSP90 (sc-13119) and murine/human Pim-2 (sc-13514) were purchased from Santa Cruz Biotechnology (Santa Cruz, CA). Antibodies against phospho-Y694-STAT5 (CS-9359), phospho-T308-Akt (CS-9275), phospho-S473-Akt (CS-9271), phospho-T246-PRAS40 (CS-2997), phospho-S939-TSC2 (CS-3615), phospho-T1462-TSC2 (CS-3617), phospho-S2481-mTOR (CS-2974), mTOR (CS-2983), phospho-T389-p70S6K (CS-9234), phospho-S65-4EBP1 (CS-9451), phospho-T37/46-4EBP1 (CS-2855), Non-phospho-4EBP1 (CS-4923), Mcl-1 (CS-5453), eIF4E (CS-9742), eIF4G (CS-2469), Pim-1 (CS-3247), human Pim-2 (CS-4730), Bim (CS-2933) were purchased from Cell Signaling (Beverley, MA). Monoclonal antibody against activated Bax (TACS-2281) or Bak (AM03) was purchased from Trevigen (Gaithersburg, MD) or Merck Millipore (Darmstadt, Germany), respectively. The Mouse IgG APC-conjugated antibody was purchased from Biotech R&D systems (Minneapolis, MN).

### Expression plasmids, transfection, and infection

The lentiviral plasmid targeting GFP or mTOR for knockdown, pLKO.1 puro GFP siRNA [52] or mTOR_1 shRNA [53], were gifts from Bob Weinberg (Addgene plasmid ^#^12273) or David Sabatini (Addgene plasmid ^#^1855), respectively. A retroviral expression plasmid for a constitutively-activated mTOR mutant, pMXs-puro-AU1-mTOR-E2419K, was constructed by subcloning the HindIII (blunted)/NotI fragment from pcDNA3-AU1-mTOR-E2419K [36], a gift from Fuyuhiko Tamanoi (Addgene plasmid ^#^19994), into the BamHI (blunted)/NotI site of the pMXs-puro vector [54], kindly provided by Dr. T. Kitamura. A retroviral expression plasmid for Pim-1, pMXs-IG-Myr-Flag-PIM1, was constructed by subcloning the EcoRI/SalI fragment from pWZL-Neo-Myr-Flag-PIM1 [55], a gift from William Hahn and Jean Zhao (Addgene plasmid ^#^20579), into the EcoRI/XhoI site of the pMXs-IG vector [54], kindly provided by Dr. T. Kitamura. The lentiviral packaging plasmid psPAX2 and the envelope expressing plasmid pMD2.G were gifts from Didier Trono (Addgene plasmid ^#^12260 and ^#^12259, respectively).

Transfection of the retroviral vectors pMXs-puro-AU1-mTOR-E2419K or pMXs-puro into PLAT-A cells and infection of 32D/ITD cells were performed as described previously [51], followed by selection with 1.0 μg/ml puromycin. 32D/TKD cells were infected with the recombinant retrovirus obtained from PLAT-A cells transfected with pMXs-IG-Myr-Flag-PIM1 or pMXs-IG, and sorted for GFP expression by flow cytometry. These cells were maintained and analyzed as described for 32D/ITD and 32D/TKD. To knock down mTOR in MV4-11 cells, these cells were infected with the recombinant lentivirus obtained from 293T cells transfected with mTOR_1 shRNA or pLKO.1 puro GFP siRNA along with psPAX2 and pMD2.G, followed by selection with 1.0 μg/ml puromycin.

### Immunoblotting, immunoprecipitation, and, cap-binding assays

Immunoblotting, immunoprecipitation, and cap-binding assays were performed essentially as described previously [21, 56], except for the following changes in lysis buffers. For detection of Pim kinases by immunoblotting, cells were lysed directly with 1 x Laemmli’s buffer. For immunoprecipitation of Bim, cells were lysed in a lysis buffer containing 1% Triton X-100, 20 mM Tris-HCl (pH 7.5), 137 mM NaCl, 1 mM EGTA, 50 mM NaF, 1.5 mM MgCl_2_, 1mM sodium orthovanadate, 10% glycerol, 1 mM phenylmethylsulfonyl fluoride and 10 μg/ml each of aprotinin and leupeptin.

### Flow cytometric analyses

Flow cytometric analyses for cell cycle and apoptosis, conformational changes of Bax and Bak, and cleavage of Caspase-3 were performed essentially as described previously [21, 57].

### Analyses of cell proliferation and viability

Cell proliferation and viability were assessed by counting viable and nonviable cell numbers by the trypan blue-dye exclusion method. Cell viability was calculated by dividing number of viable cells by that of total cells. Viable cell numbers were also assessed using the Cell counting Kit-8 (Dojindo, Japan), according to the manufacturer’s instructions. For combination studies, the synergy was assessed by the combination index (CI) of Chou and Talalay method using Compu Syn software [34]. The CI values less than 1.0 indicate synergism.

### Analyses of primary AML cells

Primary AML cells were isolated from patients and analyzed essentially as described previously [21]. The study was approved by the ethical committee of Tokyo Medical and Dental University. Written informed consent was obtained from the patient in compliance with the Declaration of Helsinki.

## SUPPLEMENTARY MATERIALS FIGURES


