# Supplementary material for: FLT3-ITD induces expression of Pim kinases through STAT5 to confer resistance to the PI3K/Akt pathway inhibitors on leukemic cells by enhancing the mTORC1/Mcl-1 pathway
Source: Oncotarget. 2017 Dec 4;9(10):8870–86. doi: 10.18632/oncotarget.22926 (PMC5823622; doi:10.18632/oncotarget.22926)
Supplement: Supplementary file 1 [file oncotarget-09-8870-s001.pdf]

FLT3-ITD induces expression of Pim kinases through STAT5 to confer resistance to the PI3K/Akt pathway inhibitors on leukemic cells by enhancing the mTORC1/Mcl-1 pathway

SUPPLEMENTARY MATERIALS

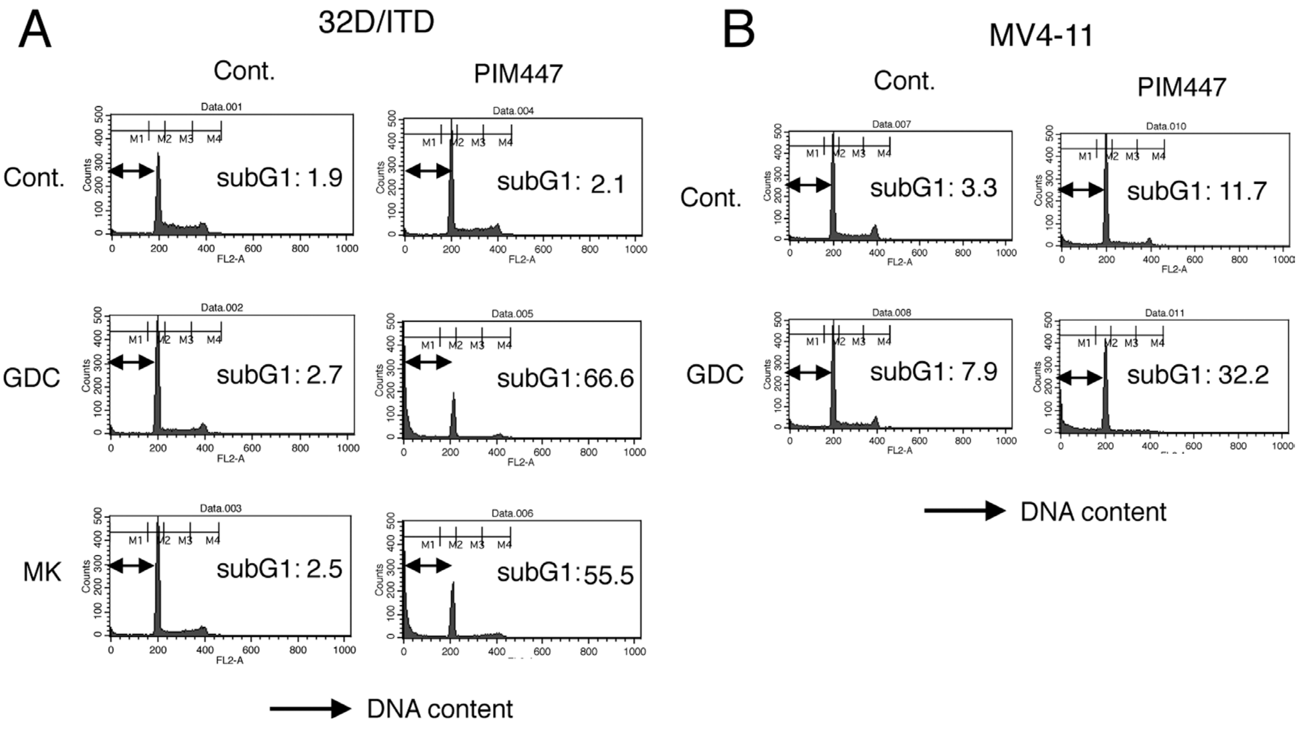

Supplementary Figure 1: The pan-Pim inhibitor PIM447 induces apoptosis synergistically with the PI3K/Akt pathway inhibitors in 32D/ITD and MV4-11 cells. (**A**) 32D/ITD cells were treated for 48 h with or without 1 μM GDC-0941 (GDC), 1 μM MK-2206 (MK), or 3 μM PIM447, as indicated, and analyzed for the cellular DNA content by flow cytometry. Percentages of apoptotic cells with sub-G1 DNA content are indicated. (**B**) MV4-11 cells were cultured for 48 h with or without 1 μM GDC-0941 (GDC) or 3 μM PIM447, as indicated, and analyzed.

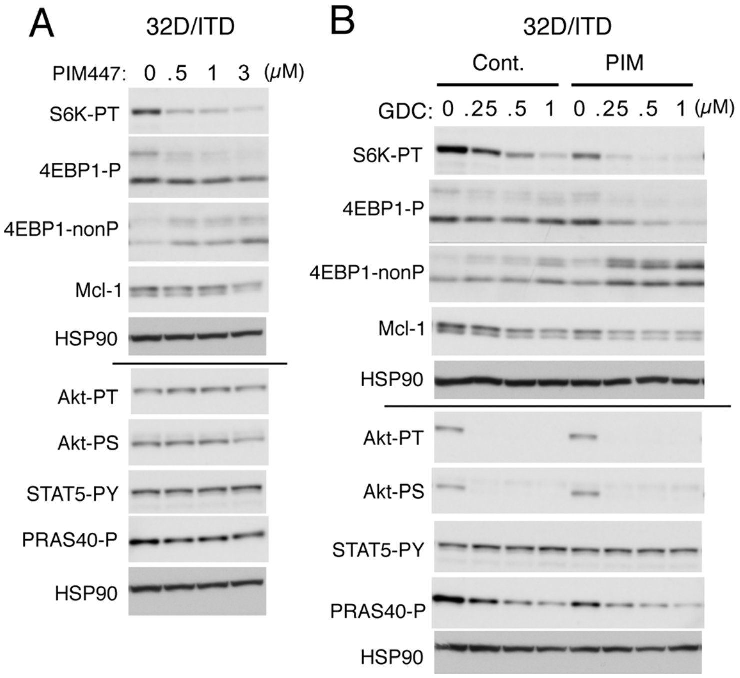

Supplementary Figure 2: The pan-Pim inhibitor PIM447 and the PI3K inhibitor GDC-0941 cooperatively downregulates the mTORC1/Mcl-1 pathway in FLT3-ITD-expressing cells. (**A**) 32D/ITD cells were cultured for 6 h with indicated concentrations of PIM447 (PIM) and lysed. Cell lysates were run on duplicate gels and subjected to Western blot analysis with antibodies against indicated proteins. The results obtained from duplicate gels are shown above or below a horizontal line. Abbreviations: S6K-PT, phospho-T389-p70S6 kinase; 4EBP1-P, phospho-T37/46-4EBP1; 4EBP1-nonP, non-phospho-T46-4EBP1; Akt-PT, phospho-T308-Akt; Akt-PS, phospho-S473-Akt; STAT5-PY, phospho-Y694-STAT5; PRAS40-P, phospho-T246-PRAS40. (**B**) 32D/ITD cells were cultured for 6 h with indicated concentrations of GDC-0941 (GDC) in the presence or absence of 1 μM PIM447, as indicated, and analyzed as in A. (**C**) MV4-11 cells were cultured for 18 h with indicated concentrations of PIM447 and analyzed. 4EBP1-S65P: phospho-S65-4EBP1. (**D**) MV4-11 cells were cultured for 18 h with 0.25 μM GDC-0941 (GDC) or 1 μM PIM447 (PIM), as indicated, and analyzed.

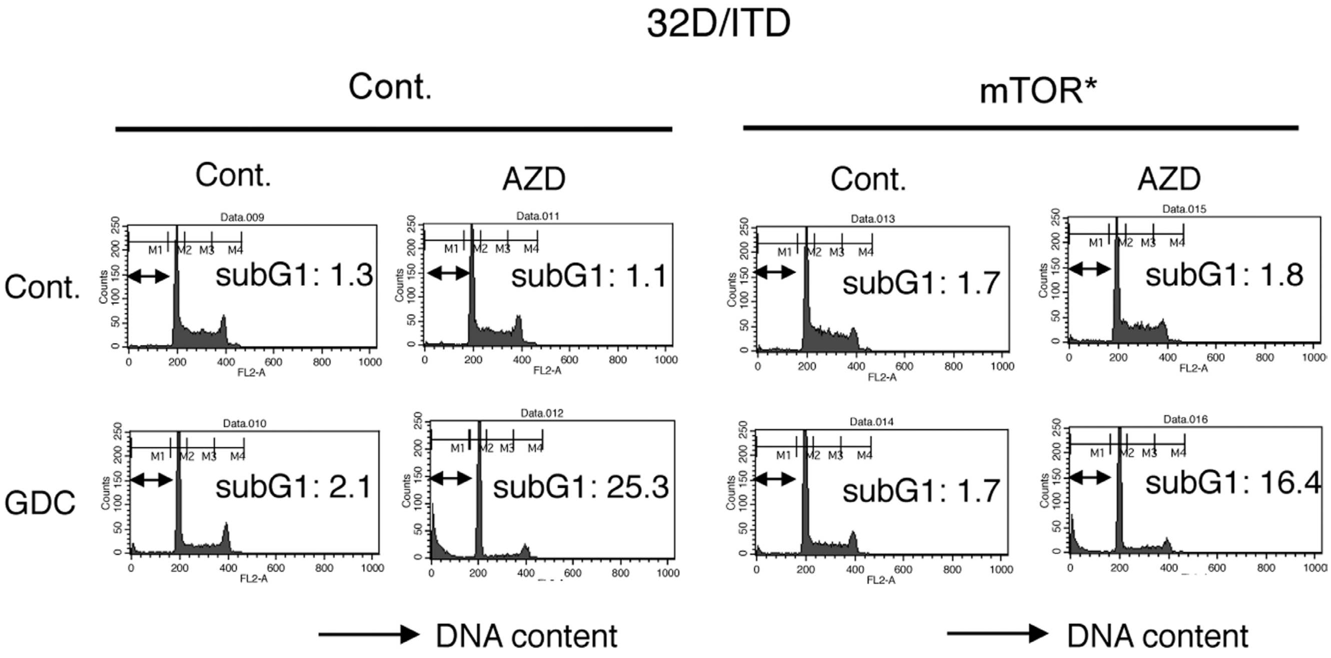

Supplementary Figure 3: Expression of the activated mTOR mutant confers on 32D/ITD cells resistance to combined treatment with GDC-0941 and AZD1208. 32D/ITD cells expressing mTOR-E2419K (mTOR*) or vector control cells (Cont.) were cultured for 48 h with or without 3 μM GDC-0941 (GDC) and 2 μM AZD1208 (AZD), as indicated, and analyzed for the cellular DNA content by flow cytometry. Percentages of apoptotic cells with sub-G1 DNA content are indicated.
